# Silver Nanoparticles-Decorated Porous Silicon Microcavity as a High-Performance SERS Substrate for Ultrasensitive Detection of Trace-Level Molecules

**DOI:** 10.3390/nano15131007

**Published:** 2025-06-30

**Authors:** Manh Trung Hoang, Huy Bui, Thi Hong Cam Hoang, Van Hai Pham, Nguyen Thu Loan, Long Van Le, Thanh Binh Pham, Chinh Vu Duc, Thuy Chi Do, Tae Jung Kim, Van Hoi Pham, Thuy Van Nguyen

**Affiliations:** 1 Institute of Materials Science, Vietnam Academy of Science and Technology, Hanoi 100000, Vietnam; trunghm@nda.org.vn (M.T.H.); buihuy@ims.vast.ac.vn (H.B.); loannt@ims.vast.ac.vn (N.T.L.); longlv@ims.vast.ac.vn (L.V.L.); binhpt@ims.vast.ac.vn (T.B.P.); chinhvd@ims.vast.ac.vn (C.V.D.); hoipv@ims.vast.ac.vn (V.H.P.); 2 Graduate University of Sciences and Technology, Vietnam Academy of Science and Technology, Hanoi 100000, Vietnam; 3 Vietnam Academy of Science and Technology, University of Sciences and Technology of Hanoi, Hanoi 100000, Vietnam; hoang-thi-hong.cam@usth.edu.vn; 4 Department of Physics, Hanoi National University of Education, Hanoi 100000, Vietnam; haipv@hnue.edu.vn; 5 Department of Physics, Thai Nguyen University of Education, Thai Nguyen University, Thai Nguyen 250000, Vietnam; chidt@tnue.edu.vn; 6 Department of Physics, Kyung Hee University, Seoul 02447, Republic of Korea

**Keywords:** microcavity, porous silicon, SERS, rhodamine 101, methyl parathion

## Abstract

In this study, we present a novel surface-enhanced Raman scattering (SERS) substrate based on porous silicon microcavities (PSiMCs) decorated with silver nanoparticles (AgNPs) for ultra-sensitive molecule detection. This substrate utilizes a dual enhancement mechanism: the localized surface plasmon resonance (LSPR) of AgNPs and the optical resonance of the PSiMC structure, which together create intense electromagnetic hot spots and prolong photon–molecule interactions. The porous architecture provides a large surface area for uniform nanoparticle distribution and efficient analyte adsorption. The AgNP/PSiMC substrate demonstrates an impressive detection limit of 1.0 × 10^−13^ M for rhodamine101 and 1.0 × 10^−10^ M for methyl parathion, outperforming many previously reported SERS platforms. Furthermore, the substrate exhibits excellent signal uniformity (RSD ≈ 6.14%) and long-term stability, retaining over 50% signal intensity after 28 days. These results underscore the potential of AgNP/PSiMCs as highly efficient, reproducible, and scalable SERS platforms for trace-level chemical and environmental sensing applications.

## 1. Introduction

Surface-enhanced Raman scattering (SERS) has emerged as a powerful spectroscopic technique that significantly enhances the inherently weak Raman signals of analyte molecules through the interaction with nanostructured metallic surfaces. This enhancement occurs when analytes are positioned close to, or directly adsorbed onto, the rough surfaces of noble metal nanoparticles such as silver, gold, or copper. Due to its exceptional sensitivity, SERS has been extensively applied in diverse fields including biosensing, food safety, pharmaceutical analysis, explosive detection, and environmental monitoring [1,2,3,4]. The remarkable sensitivity of SERS, with reported enhancement factors reaching up to 14 orders of magnitude, is primarily attributed to localized surface plasmon resonance (LSPR). This phenomenon arises from the interaction between incident laser light and the collective oscillations of conduction electrons in metal nanoparticles [5,6]. The resulting plasmonic excitation creates highly confined electromagnetic fields, enabling the detection of molecular signatures down to the single-molecule level [7,8].

The performance of SERS is highly dependent on the properties of the substrate, sensing mechanism, and the surrounding environment. To optimize sensitivity, nanostructured substrates are commonly employed due to their large specific surface area and enhanced adsorption capabilities. Among these, porous silicon (PSi) materials have gained increasing attention due to their high surface area, tunable morphology, biocompatibility, and compatibility with existing silicon-based fabrication technologies [9]. PSi is a sponge-like silicon material produced via electrochemical etching, where the silicon wafer serves as the anode. This method is simple, cost-effective, and highly controllable. By adjusting the anodization current density, the porosity of the silicon can be tuned, while etching time controls the pore depth. Additionally, the pore diameter and surface morphology can be further tailored by changing the electrolyte composition, etching parameters, and the crystalline orientation or doping type of the silicon wafer [10,11,12]. Due to its flexibility, PSi has been widely applied in areas such as biosensors [13], solar cells [14], biomedical devices [9], and catalysis [15]. In recent years, PSi-based SERS substrates have also been extensively investigated [16,17,18], where noble metal nanoparticles (Au, Ag, or alloys) are deposited onto the porous silicon matrix to create high-density electromagnetic hotspots, resulting in excellent SERS performance [19,20,21,22].

The ability to precisely modulate the refractive index through porosity control makes PSi a highly suitable material for fabricating one-dimensional (1D) photonic structures, including rugate filters [23], Bragg reflectors [24], and microcavities [25], which exhibit useful light-trapping and interference properties. In particular, porous silicon microcavities (PSiMCs), consisting of two distributed Bragg reflector (DBR) mirrors sandwiching a cavity layer of optical thickness λ or λ/2, have attracted attention for photonic and sensing applications. The DBRs are formed by stacking alternating layers of high and low porosity, creating a one-dimensional photonic crystal structure with strong optical interference effects. The central spacer layer acts as a resonant cavity, where constructive interference enhances light–matter interaction within the localized optical field. When functionalized with silver nanoparticles (AgNPs), PSiMC structures combine plasmonic enhancement and photonic confinement, forming a hybrid platform with superior SERS sensitivity. Despite the strong potential of this design, SERS substrates based on metal-decorated PSiMCs remain largely underexplored. Most studies have focused on single-layer PSi [26,27] or surface-modified porous structures [28,29,30]; in contrast, SERS sensors utilizing PSiMC structures have been rarely reported.

In this study, we present a novel surface-enhanced Raman scattering (SERS) platform based on porous silicon microcavities (PSiMCs) functionalized with silver nanoparticles (AgNPs). This hybrid structure harnesses the synergistic benefits of photonic resonance within the microcavity and localized surface plasmon resonance (LSPR) from the AgNPs, resulting in the formation of densely distributed electromagnetic “hotspots” that dramatically amplify Raman signals. The substrate is fabricated through a simple, cost-effective, and scalable electrochemical process that is fully compatible with standard silicon processing technologies. Using rhodamine 101 (Rh101), a xanthene dye featuring dual chromophores that facilitate resonance enhancement, we systematically demonstrate the platform’s high sensitivity, signal uniformity, and stability. Additionally, the SERS spectra of Rh101 obtained from PSi single-layer and photonic crystal substrates serve as references. Furthermore, the detection of methyl-parathion (MP), a hazardous environmental contaminant, underscores the potential of AgNP/PSiMCs as a powerful and reproducible SERS substrate for label-free trace detection in environmental safety, food monitoring, and biosensing applications.

## 2. Materials and Methods

### 2.1. Materials

The monocrystalline silicon wafers used in this investigation were p-type, boron-doped, with a typical resistivity of 0.01–0.013 Ω·cm, (100) orientation, and a thickness of 625 µm, sourced from CrysTec GmbH, Germany. The electrolyte solution consisted of 48 wt% hydrofluoric acid (HF, Merck Millipore, Germany) and 99.9% absolute ethanol (Merck Millipore) in a 1:2 volume ratio. Silver nitrate (AgNO_3_, ≥99.0%, Sigma-Aldrich) was dissolved in deionized (DI) water at a concentration of 10^−2^ M. Rhodamine 101 (Sigma-Aldrich, Germany) was also dissolved in water to prepare a standard stock solution at a concentration of 10^−2^ M. Different concentrations (10^−7^ M to 10^−13^ M) were prepared by successive dilutions of the stock solution with water. Methyl parathion (MP) purchased from Sigma-Aldrich was first dissolved in a small amount of acetone to prepare a concentrated stock solution at 10^−1^ M. This stock solution was subsequently diluted with deionized water to prepare analyte solutions at concentrations ranging from 10^−6^ M to 10^−10^ M. Analyte solutions (10 µL each) were dropped onto the AgNP/PSiMC surface and air-dried to form samples for Raman spectroscopic measurements.

### 2.2. Preparation of Porous Silicon Substrates

The PSi samples used in this experiment were fabricated using anodic electrochemical etching in an electrolyte solution composed of 48% HF and absolute ethanol mixed in a 1:2 (*v*/*v*) ratio. The PSi multilayer structure, also known as a distributed Bragg reflector (DBR), was designed by periodically modulating the current density during the etching process, creating alternating layers of low and high refractive indices. This configuration consists of a total of 12 layer pairs. The PSi microcavity resonator structure comprises two DBR stacks separated by a spacer layer with a thickness of either λ or λ/2. The bottom DBR contains more periods than the top DBR to compensate for the additional losses that arise as a function of depth in the silicon. Detailed etching parameters for all structures are provided in Table 1. Notably, a monolayer PSi structure was fabricated using a current density of 50 mA/cm^2^, corresponding to the refractive index of the low-index layer in the DBR configuration.

### 2.3. Fabrication of SERS Substrates

Silver nanoparticles (AgNPs) were deposited onto PSiMC substrates using an immersion plating technique. This method takes advantage of the high density of Si-H bonds on the porous silicon surface, which facilitates the reduction of Ag^+^ ions to metallic silver without the need for additional chemical reducing agents. As a result, AgNPs can be synthesized in a clean, straightforward process that avoids the use of toxic chemicals. Immersion plating is therefore a widely used and convenient approach for generating AgNPs on solid surfaces. The density and average size of AgNPs are closely dependent on parameters such as dipping time and the concentration of the AgNO_3_ solution. In this study, five types of samples were obtained by immersing the substrates for varying durations: 25, 50, 75, 100 and 125 s in 0.01 M AgNO_3_ solution. After immersion, the PSiMC samples were left to dry naturally in the air and were utilized as SERS sensor probes for detecting rhodamine 101 (Rh101) and methyl parathion (MP) pesticide residues.

### 2.4. Characterizations and SERS Measurement

The crystalline structure of the AgNP/PSiMC composites was analyzed using a high-resolution X-ray diffractometer (XRD), specifically the D8-Advance model, which features a powder diffraction setup and employs a Cu Kα radiation source (λ = 1.5406 Å). To investigate the surface morphology and structural characteristics of both the as-prepared PSiMC and AgNP/PSiMC samples, a field-emission scanning electron microscope (FESEM, Hitachi 4800, Japan) was utilized. The elemental composition was further examined through energy-dispersive X-ray spectroscopy (EDX), which is integrated into the FESEM system. For optical characterization, reflectance spectra of PSiMC and AgNP/PSiMC substrates were measured using an Ocean Optics USB-4000 spectrometer paired with an HL-2000 halogen light source from the same manufacturer.

SERS measurements were performed using a LabRAM HR Evolution Raman microscope system from Horiba Scientific, equipped with a 785 nm excitation laser. The laser spot size on the sample surface was approximately 1 µm in diameter. Analytical standard solutions were created using varying concentration gradients, and 10 µL of these analyte solutions were drop cast onto the AgNP/PSiMC and left to dry naturally overnight under ambient conditions.

## 3. Results and Discussion

### 3.1. Structure and Morphological Characteristics

**Figure 1:**
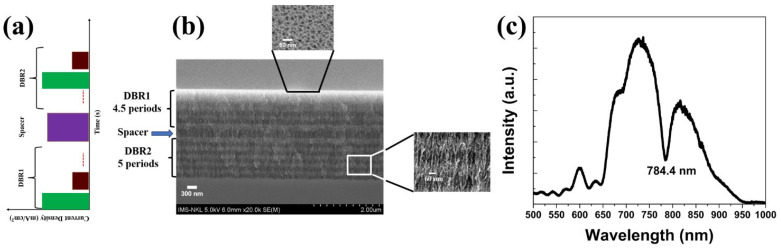
(**a**) Schematic of the microcavity structure formed via time-modulated current density during electrochemical etching. (**b**) Cross-sectional SEM image showing alternating porosity regions and a central cavity; inset images show zoomed-in views of the surface and cross-sectional pore morphology. (**c**) Reflectance spectrum displaying a sharp resonance at 784.4 nm, confirming optical confinement within the cavity.

Figure 1 presents the schematic design, structural morphology, and optical characterization of a porous silicon microcavity (PSiMC) fabricated via electrochemical etching. As illustrated in Figure 1a, the microcavity consists of alternating layers of high and low porosity silicon arranged symmetrically around a central optical cavity. This architecture is realized by modulating the etching current density over time, enabling precise control over porosity and, consequently, the refractive index profile throughout the depth of the structure. The cross-sectional SEM image in Figure 1b confirms the successful formation of the PSiMC. The alternating bright and dark layers correspond to the modulation of porosity, which forms the photonic crystal-like structure. The central cavity, clearly visible as a disruption in periodicity, serves as the optical resonance site. Inset images in Figure 1b provide zoomed-in views of both the top surface morphology, showing a nanoporous texture, and the vertical pore structure within the multilayer, which confirms the anisotropic and vertically aligned nature of the etching process. The reflectance spectrum shown in Figure 1c reveals a distinct and narrow resonance dip at 784.4 nm, corresponding to the optical mode confined within the central cavity. This resonance is located within the photonic bandgap formed by the periodic porosity layers, validating the optical functionality of the microcavity. The close alignment of this resonance with common Raman excitation wavelengths (e.g., 785 nm) highlights the suitability of the structure for optical sensing applications, including cavity-enhanced Raman scattering. Together, these structural and optical characterizations demonstrate that the fabricated PSiMC is a well-defined, optically resonant platform capable of confining light within the infrared–visible range.

**Figure 2:**
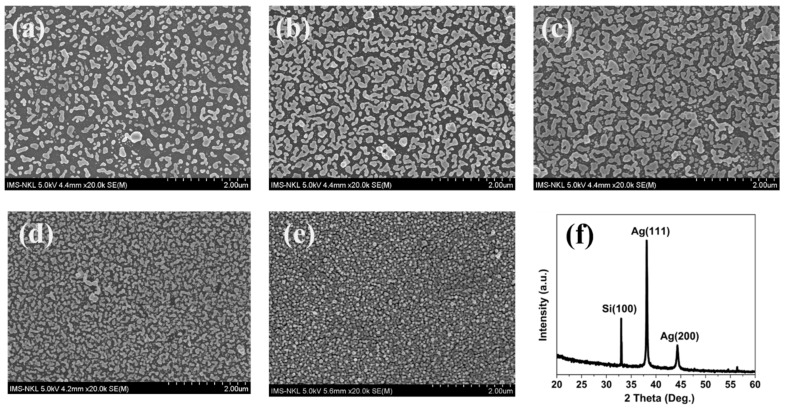
(**a**–**e**) Surface topographies of silver nanoparticle (AgNP)-decorated PSiMC substrates with immersion times of 25, 50, 75, 100 and 125 s, respectively and (**f**) XRD spectrum of AgNPs deposited on the PSiMC surface with deposition time of 75 s.

Figure 2 illustrates the evolution of the surface morphology of silver nanoparticles (Ag NPs) formed on freshly etched PSiMC substrates by immersing them in a 0.01 M AgNO_3_ solution at the following time intervals: 25 s, 50 s, 75 s, 100 s, and 125 s. At short deposition times of 25 s and 50 s (Figure 2a,b), AgNPs are sparsely and irregularly distributed. The nucleation process is driven by the high density of surface Si-H bonds in freshly etched PSi, resulting in particles ranging from sub-micron to nanometer sizes. However, the coverage remains incomplete, and the size distribution is broad. At 75 s (Figure 2c), the AgNPs achieve an optimal distribution for plasmonic enhancement, exhibiting a uniform size of 30–40 nm, with interparticle distances of 5–20 nm. This arrangement forms semi-continuous yet well-separated nanoparticle networks [31], maximizing the density of plasmonic “hot spots” and providing ideal conditions for strong LSPR-based SERS enhancement. Additionally, the mesoporous structure allows smaller AgNPs to embed within the pores, while larger particles anchor to the surface. The particle size and spacing were estimated from SEM images using ImageJ (version 1.54k) software by measuring multiple representative particles per sample. With longer deposition times of 100 s and 125 s (Figure 2d,e), excessive growth leads to the coalescence of Ag NPs, forming larger clusters and increased surface coverage. This morphological transition decreases nanogap density and weakens electromagnetic field concentration, potentially compromising SERS performance. The X-ray diffraction (XRD) pattern in Figure 2f, corresponding to the 75 s deposition sample, shows prominent diffraction peaks at 2θ ≈ 38.1° (Ag(111)) and 44.3° (Ag(200)), confirming the formation of crystalline silver with a face-centered cubic structure. The strong Ag(111) peak indicates a preferential orientation, consistent with thermodynamically favored growth while a weaker Si(100) peak is also visible, corresponding to the underlying silicon substrate.

**Figure 3:**
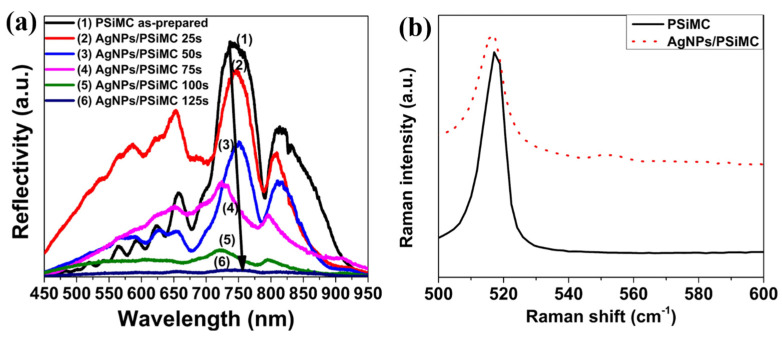
(**a**) Reflectivity spectra of porous silicon microcavity (PSiMC) before and after silver nanoparticle (AgNP) decoration at various immersion times and (**b**) Raman pattern of PSiMC before and after AgNP decoration (at 75 s).

The optical response of the Ag-decorated PSiMC nanostructures was evaluated through specular reflectance measurements. Figure 3a illustrates the changes in reflectance spectra corresponding to Ag deposition times ranging from 25 to 125 s. As the deposition time increased, the cavity resonance became broader and less defined. This behavior can be attributed to the increased surface coverage by AgNPs, which disrupt the periodicity necessary for strong optical interference. Additionally, the results suggest that silver nanoparticles may infiltrate into the porous silicon matrix during longer deposition times. Notably, interference fringes remained clear for deposition times up to 75 s; however, at 100 s, the reflectance spectrum showed an almost complete loss of interference patterns. These findings are consistent with the surface structural changes observed in the SEM analysis. Figure 3b presents the Raman spectra of PSiMC samples before and after decoration with AgNPs. In both samples, a strong Raman peak is centered at approximately 520 cm^−1^, corresponding to the first-order optical phonon mode of crystalline silicon (c-Si). The bare PSiMC sample (black curve) shows a symmetric and sharp Raman peak, indicating minimal stress and uniform nanostructure within the as-prepared porous silicon. After AgNP decoration (red dashed curve), the Raman peak broadens significantly compared with the bare PSiMC sample. The peak broadening can be attributed to two main effects: (i) the size distribution broadening of the silicon nanostructures due to surface modifications [32], and (ii) a local temperature increase caused by plasmonic heating from the excitation of AgNPs [33]. Plasmonic nanoparticles are known to enhance local electromagnetic fields and can induce localized photothermal effects during Raman excitation, leading to phonon softening and broader Raman features.

### 3.2. SERS Evaluation of AgNP-Coated PSiMC Active Substrates

**Figure 4:**
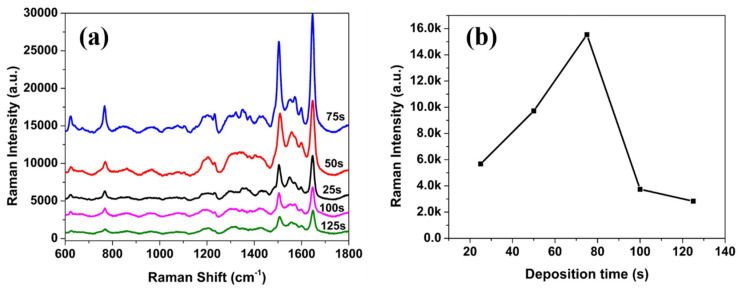
(**a**) Surface-enhanced Raman spectra of Rhodamine 101 (1.0 × 10^−9^ M) recorded on AgNP/PSiMC substrates fabricated with different Ag deposition times (25, 50, 75, 100, and 125 s) and (**b**) dependence of the intensity of 1646 cm^−1^ band of Rh101 on the deposition time.

The SERS performance was first examined and evaluated using Rh101 dye as the target analyte. Figure 4a shows the SERS spectra of Rh101 (1.0 × 10^−9^ M) acquired from AgNP/PSiMC substrates with varying silver deposition durations in 0.01 M AgNO_3_ solution. The Raman signals progressively strengthen from 25 to 75 s, with a dramatic enhancement observed at 75 s. Prominent peaks, particularly around 1646 cm^−1^, characteristic of aromatic C=C stretching in the xanthene ring of Rh101, demonstrate the sensitivity of the plasmonic-active PSiMC substrates. This enhancement can be attributed to the optimized size and interparticle gap of silver nanoparticles at 75 s, which results in maximal localized surface plasmon resonance (LSPR) and the formation of abundant electromagnetic “hot spots.” Such conditions greatly amplify the incident and scattered Raman fields near the adsorbed Rh101 molecules. However, as the silver deposition time increases further to 100 and 125 s, the overall Raman signal declines. This attenuation is commonly linked to Ag nanoparticle overgrowth and aggregation, which reduces the effective hot spot density due to increased interparticle distances and loss of nanogap uniformity. The results correlate with the SEM observations shown in Figure 2a–e, indicating that longer immersion times lead to larger and more irregular AgNPs formations. Additionally, excessive surface coverage may lead to shadowing effects or quenching phenomena that limit light–analyte interaction [34]. Figure 4b quantifies the trend by plotting the intensity of the ~1646 cm^−1^ band against deposition time. The observed dependence indicates that the AgNP morphology at 75 s offers the most favorable nanostructure for SERS enhancement in detecting Rh101 at trace concentrations. These findings underscore the critical importance of controlling AgNP deposition kinetics when engineering nanostructured SERS substrates for the efficient detection of fluorescent dyes such as Rh101. Table 2 illustrates the characteristic Raman peaks of Rh101. The intensity of the peak at 1646 cm^−1^ was selected as the parameter by which to characterize the SERS signals.

**Figure 5:**
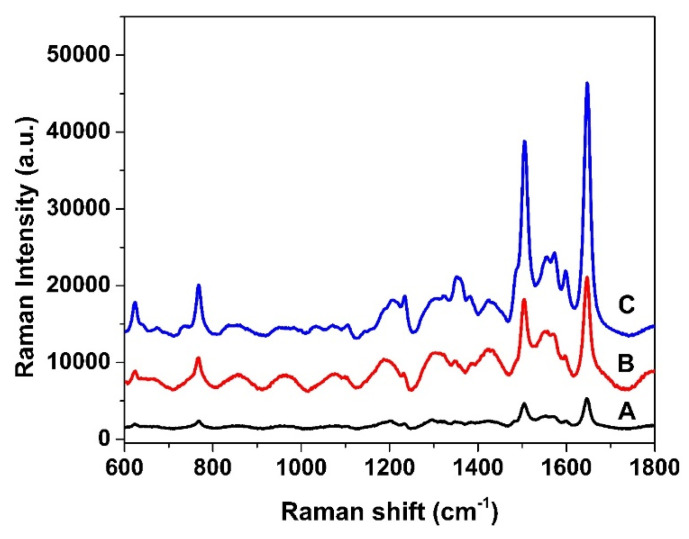
Raman spectra of Rh101 (1.0 × 10^−7^ M) detected on (A) PSi single-layer; (B) photonic crystal and (C) microcavity substrates after AgNP deposition. The substrates were treated in 0.01 M AgNO_3_ solution for the 75 s.

Figure 5 illustrates the observed variation in Raman intensity among the three PSi-based substrates: single-layer (A), photonic crystal (B), and microcavity (C). This variation is primarily attributed to differences in the surface electric field distribution after AgNP deposition. It is well established that photonic crystal structures, due to their periodic multilayer configuration, can support photonic band-gap effects and spatially confine the optical field near the surface [38,39]. This optical field confinement enhances the localized surface plasmon resonance (LSPR) effect when noble metal nanoparticles are present, resulting in stronger electromagnetic enhancement and improved SERS performance. Compared with the single-layer substrate, the photonic crystal substrate exhibits significantly stronger Raman signals, despite comparable porosity in their low-index layers. This suggests that the enhancement is primarily due to electromagnetic effects rather than differences in molecular adsorption. Moreover, the microcavity structure, which introduces a resonant cavity within the photonic crystal framework, demonstrates the highest Raman intensities [40,41]. This can be attributed to further enhancement of light–matter interactions and the formation of highly localized electromagnetic “hot spots” at the AgNP interface. Therefore, these results confirm the critical role of surface field localization and optical confinement in enhancing SERS signals and underscore the superiority of microcavity and photonic crystal architectures in developing high-sensitivity SERS-active substrates.

**Figure 6:**
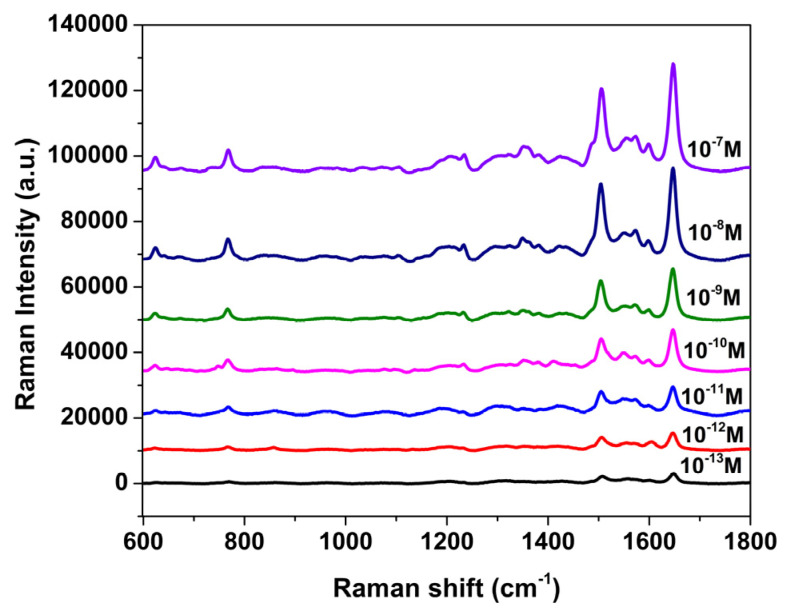
Raman spectra of Rh101 (10^−13^ M–10^−7^ M) on AgNP/PSiMC substrates. PSiMC samples were treated in 0.01 M AgNO_3_ solution for 75 s.

The detection limit is one of the key parameters used to evaluate the performance of a SERS-active substrate, as it reflects the lowest analyte concentration at which a Raman signal can be reliably detected. To characterize the sensitivity of the AgNP/PSiMCsubstrates, Rh101 was used as the probe molecule in a concentration range from 1.0 × 10^−7^ M to 1.0 × 10^−13^ M. PSiMC samples were immersed in 0.01 M AgNO_3_ aqueous solution for 75 s prior to measurement. As shown in Figure 6, a gradual increase in Raman intensity was observed as the Rh101 concentration increased. Importantly, distinguishable characteristic peaks of Rh101, particularly near 1350 cm^−1^ and 1648 cm^−1^, remained detectable above the noise level at concentrations as low as 1.0 × 10^−13^ M, indicating the high sensitivity of the AgNP/PSiMC substrate. In this work, the detection limit refers to the lowest Rh101 concentration that yielded a signal-to-noise ratio (S/N) greater than 3 [42,43,44]. These results demonstrate the remarkable sensitivity of the PSiMC-based SERS platform and its suitability for ultra-trace molecular detection in future chemical or biosensing applications.

Quantifying the signal enhancement in SERS typically involves calculating the enhancement factor (*EF*), which requires knowledge of both the Raman intensity and the number of analyte molecules within the laser excitation volume as the following formula [28]:

(1) $$EF=\frac{I_{Ag/PSi}/N_{Ag/PSi}}{I_{Si}/N_{Si}}$$

where *I* refers to the measured Raman signal intensity and *N* represents the quantity of Rh101 molecules present within the region exposed to the laser beam. The notation *Ag/PSi* corresponds to a PSiMC substrate modified with AgNPs, whereas *Si* indicates an unmodified silicon substrate.

For porous silicon substrates, determining the exact number of analyte molecules within the irradiated area is practically difficult due to the complex internal structure. As a result, Equation (1) is not applicable for directly calculating the enhancement factor of AgNP/PSiMC substrates. To address this, a widely used alternative, external amplification Raman efficiency, is employed [34]. This method evaluates the enhancement by calculating the ratio between the minimum detectable concentration of analyte on the Ag-coated PSiMC substrate and that of a bare Si substrate. For flat Si substrates, the lowest detectable concentration of Rh101 using the immersion method is approximately 1.0 × 10^−2^ M. In comparison, on the AgNP/PSiMC substrate, Rh101 can be detected down to 1.0 × 10^−13^ M, as confirmed by the experimental results. Therefore, the amplification Raman efficiency for the AgNP/PSiMC substrate is 10^11^.

**Figure 7:**
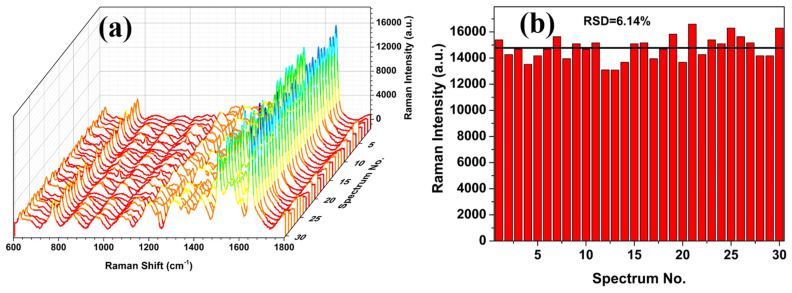
(**a**) Raman spectra of Rh101 (10^−9^ M) adsorbed on AgNP/PSiMC substrates, recorded at thirty randomly selected positions across the sample surface. (**b**) The line indicates the average SERS intensity of the 30 random positions at peak of 1646 cm^−1^ with RSD = 6.14%.

To assess the reproducibility of the AgNP/PSiMC substrate, SERS spectra of Rh101 (10^−9^ M) were collected from thirty randomly selected positions from three independently prepared substrates, all measured under the same experimental conditions. As shown in Figure 7, the SERS signals exhibited similar intensity, demonstrating the uniformity of SERS enhancements across the entire surface of the AgNP/PSiMC substrates. The calculated relative standard deviation (RSD) of 6.14% at the 1646 cm^−1^ peak confirms good signal uniformity, indicating reliable fabrication and homogenous AgNP deposition. This level of reproducibility highlights the substrate’s potential for practical SERS applications that require both sensitivity and consistency.

**Figure 8:**
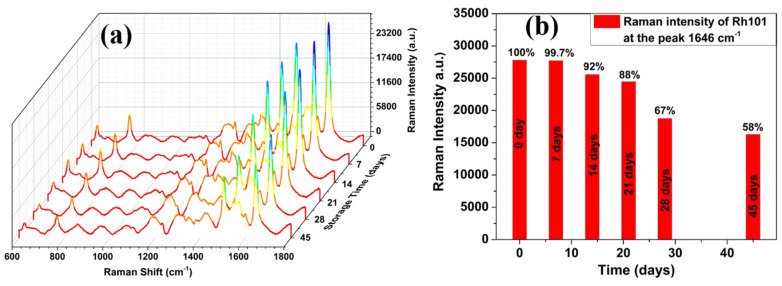
(**a**) Raman spectra of Rh101 (10^−8^ M) adsorbed on AgNP/PSiMC substrates within 45 days, (**b**) the relationship between the SERS intensity at 1646 cm^−1^ and storage time.

The long-term stability of the AgNP/PSiMC SERS substrate was evaluated through a systematic aging test. Six samples were fabricated following the same protocol. One freshly prepared sample was used to adsorb Rh101 10^−8^ M directly, while the remaining five were stored in a vacuum desiccator, protected from light, for 7, 14, 21, 28, and 45 days, respectively. After each storage period, the aged samples were treated with Rh101 under the same conditions as the fresh one, and their Raman spectra were recorded. As shown in Figure 8a, the characteristic Raman peak at 1646 cm^−1^ remained visible across all storage durations, although a gradual decline in intensity was observed over time. Figure 8b presents the intensity variation at this peak, with the substrate maintaining approximately 99.7%, 92%, 88%, 67%, and 58% of the initial signal intensity after 7, 14, 21, 28, and 45 days, respectively. Notably, the Raman signal retained more than 50% of its original intensity even after four weeks of storage. These results confirm that the AgNP/PSiMC substrate exhibits excellent storage stability and reproducibility, making it a reliable platform for practical SERS applications in trace-level detection.

### 3.3. Detection of Methyl Parathion on SERS Active Substrate

Methyl parathion (MP) is a highly toxic organophosphorus pesticide widely used in agriculture to control insect pests. Despite its effectiveness, its persistence in the environment and strong neurotoxicity pose significant threats to human health and ecological safety. MP can accumulate in soil, water sources, and agricultural produce, including vegetables and fruits, entering the food chain and potentially leading to chronic exposure. Prolonged contact with trace levels of this compound has been linked to severe health effects, including disruption of the nervous system, reproductive toxicity, and carcinogenic risks. In this work, we utilize AgNP/PSiMC substrates as an effective SERS-active platform for detecting MP residues.

**Figure 9:**
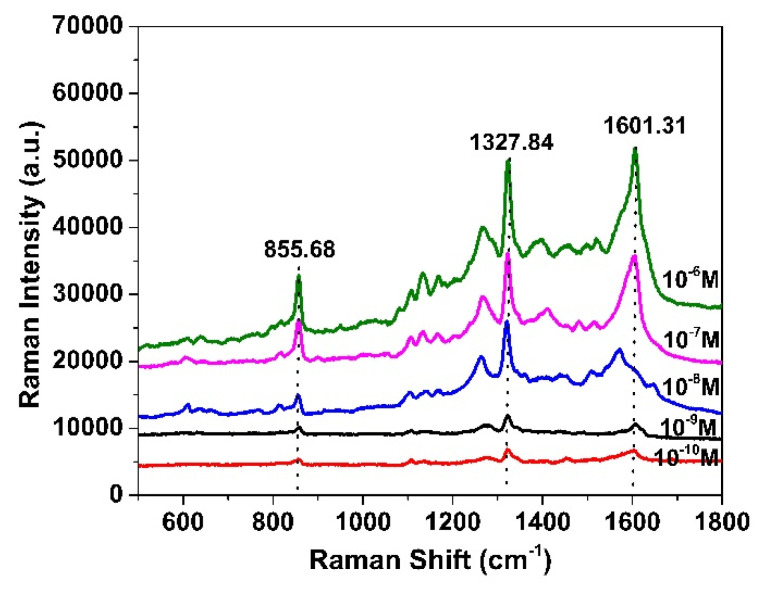
Raman spectra of methyl parathion with concentrations ranging from 10^−10^ M to 10^−6^ M using AgNP/PSiMC substrate.

Figure 9 displays the Raman spectra of MP measured on the AgNP/PSiMC substrate across a series of concentrations ranging from 1.0 × 10^−6^ M to 1.0 × 10^−10^ M. As the concentration decreased, a consistent decline in Raman signal intensity was observed, confirming the substrate’s ability to capture variations in analyte concentration. Three prominent peaks located at 855.68 cm^−1^, 1327.84 cm^−1^, and 1601.31 cm^−1^ were clearly distinguishable at all tested concentrations, corresponding to the NO_2_ bending, P=O and CH_3_ stretching, and C=C aromatic ring stretching vibrations, respectively [45,46,47]. Notably, even at the lowest concentration of 10^−10^ M, the peak at 1327.84 cm^−1^ remained clearly identifiable above the noise level, satisfying the SERS criterion of a signal-to-noise ratio (S/N) > 3. This result confirms the excellent sensitivity of the AgNP/PSiMC platform for trace detection of MP, achieving detection down to the sub-nanomolar level. The clear and reproducible spectral features across multiple orders of magnitude further demonstrate the strong enhancement capability and reproducibility of the developed SERS substrate.

Table 3 presents a comparison of detection limits, excitation wavelengths, and Raman systems used in recent studies on methyl parathion detection. Among these, the AgNP/PSiMC substrate demonstrated a superior detection limit of 1.0 × 10^−10^ M, surpassing or matching several state-of-the-art nanostructured SERS platforms. This enhanced sensitivity is attributed to the strong localized electromagnetic field and effective plasmonic confinement within the microcavity structure. These results highlight the AgNP/PSiMC substrate as a promising SERS platform for ultra-trace detection of pesticide residues in food safety and environmental monitoring.

## 4. Conclusions

We have successfully developed a highly sensitive and reproducible SERS substrate by integrating silver nanoparticles onto porous silicon microcavity (PSiMC) structures. The synergistic interaction between plasmonic AgNPs and photonic confinement within the microcavity architecture significantly enhances Raman signal intensity and detection performance. The AgNP/PSiMC substrate demonstrated excellent sensitivity toward Rhodamine 101, achieving a detection limit as low as 1.0 × 10^−13^ M, and enabled reliable detection of the organophosphorus pesticide methyl-parathion down to 1.0 × 10^−10^ M. In addition to its high analytical sensitivity, the platform exhibited superior signal reproducibility (RSD of 6.14%) and good long-term stability. These findings establish the AgNP/PSiMC substrate as a promising SERS platform for environmental monitoring, food safety assessment, and the detection of hazardous trace compounds in complex matrices.

## Figures and Tables

**Table 1:** Electrochemical etching conditions for PSi structures.

Sample		Current Density (mA/cm^2^)	Time (sec)	Thickness
Single layer	Low index layer	50	93	2.9 µm
DBR (12 periods)	High index layer	15	8.4	126.6 nm
	Low index layer	50	3.2	112.6 nm
PSiMC	Top DBR (4.5 periods)	15	8.4	126.6 nm
		50	3.2	112.6 nm
	Spacer	50	6.4	225.2 nm
	Bottom DBR (5 periods)	15	8.4	126.6 nm
		50	3.2	112.6 nm

**Table 2:** Assignment of Raman bands in SERS spectra of Rh101 [35,36,37].

Raman Shift (cm^−1^)	Assignment	Raman Shift (cm^−1^)	Assignment
622	C-C-C ring in-plane vibration	1232	CH_2_ + CH wag + C-O-C stretch
673	C-C-C ring out-of-plane vibration	1347	C-O-C stretch + C-C-C ring stretch
768	C-H out-of-plane bending	1423	
846	CH_2_ out-of-plane wag	1504	CH_2_ scissoring + C-N stretch
962	CH_2_ + CH wag + C-C-C ring vibration + C-O-C stretch	1570	Aromatic C-C stretching
1061	CH_2_ + CH wag + C-C-C ring vibration	1597	Xanthene ring stretch
1203	CH_2_ + CH wag + C-O-C stretch	1646	Aromatic C-C stretching

**Table 3:** Summary of SERS substrates applied for methyl parathion detection using various Raman systems.

SERS Substrate Type	Raman Instrumentation	Excitation Wavelength (nm)	Reported Detection Limit	Reference
Ag nanocube/graphene oxide composite	Renishaw confocal microprobe Raman (India)	532	2.0 × 10^−12^ M	[48]
Au nanorods sensor	Ocean Optics handheld Raman	785	1.0 × 10^−6^ M	[49]
Ag–ZnO nanorods with PAN-nanopillar architecture	Renishaw inVia confocal micro-Raman	532	1.0 × 10^−8^ M	[50]
AuNP-based pseudo-paper film (APPF)	Portable Raman (B&W Tek)	785	~5.1 × 10^−11^ M	[51]
Au@Ag core–shell nanoparticles	Confocal Raman system	633	~3.8 × 10^−9^ M	[52]
AuNP–analyte/AuNP double-decker structure	Portable Raman spectrometer (QE Pro, Ocean Optics)	785	1.0 × 10^−8^ M	[47]
Filter paper coated with AgNPs	B&W Tek portable Raman	785	~5.5 × 10^−8^ M	[45]
AgNP/PSi microcavity (PSiMC)	Confocal Raman microscope (Horiba LabRAM)	785	1.0 × 10^−10^ M	This work

## Data Availability

The data presented in this study are available on request from the corresponding author.
